# The Methodological Quality and Challenges in Conducting Economic Evaluations of Newborn Screening: A Scoping Review

**DOI:** 10.3390/ijns6040094

**Published:** 2020-11-23

**Authors:** Pasquale Cacciatore, Laurenske A. Visser, Nasuh Buyukkaramikli, Catharina P. B. van der Ploeg, M. Elske van den Akker-van Marle

**Affiliations:** 1 Sezione di Igiene, Istituto di Sanità Pubblica, Università Cattolica del Sacro Cuore, 00168 Rome, Italy; pasqualecacciatore@gmail.com; 2 Erasmus School of Health Policy & Management, Erasmus University Rotterdam, 3062 PA Rotterdam, The Netherlands; l.a.visser@eshpm.eur.nl (L.A.V.); nasuhcagdas@gmail.com (N.B.); 3 Department of Child Health, TNO, 2316 ZL Leiden, The Netherlands; kitty.vanderploeg@tno.nl; 4 Unit Medical Decision Making, Department of Biomedical Datasciences, Leiden University Medical Center, 2333 ZA Leiden, The Netherlands

**Keywords:** economic evaluations, newborn screening, decision analysis, methodology

## Abstract

Introduction: Cost-effectiveness (CEA) and cost–utility analyses (CUA) have become popular types of economic evaluations (EE) used for evidence-based decision-making in healthcare resource allocation. Newborn screening programs (NBS) can have significant clinical benefits for society, and cost-effectiveness analysis may help to select the optimal strategy among different screening programs, including the no-screening option, on different conditions. These economic analyses of NBS, however, are hindered by several methodological challenges. This study explored the methodological quality in recent NBS economic evaluations and analyzed the main challenges and strategies adopted by researchers to deal with them. Methods: A scoping review was conducted according to PRISMA methodology to identify CEAs and CUAs of NBS. The methodological quality of the retrieved studies was assessed quantitatively using a specific guideline for the quality assessment of NBS economic evaluations, by calculating a general score for each EE. Challenges in the studies were then explored using thematic analysis as a qualitative synthesis approach. Results: Thirty-five studies met the inclusion criteria. The quantitative analysis showed that the methodological quality of NBS economic evaluations was heterogeneous. Lack of clear description of items related to results, discussion, and discounting were the most frequent flaws. Methodological challenges in performing EEs of neonatal screenings include the adoption of a long time horizon, the use of quality-adjusted life years as health outcome measure, and the assessment of costs beyond the screening interventions. Conclusions: The results of this review can support future economic evaluation research, aiding researchers to develop a methodological guidance to perform EEs aimed at producing solid results to inform decisions for resource allocation in neonatal screening.

## 1. Introduction

Since the first broad-based test for phenylketonuria in the 1960s, newborn screening tests (NBS) have become a popular practice to detect potential, treatable diseases during the first days of neonatal life [1]. Thus, the main goal of NBS is to treat affected newborns before symptoms become apparent, to prevent negative effects due to late detection of the disease, and increase health outcomes [2]. Neonatal screening can refer to individual tests (such as a hearing screen or pulse oximetry) or to a whole range of analyses carried out on neonatal blood spots (test panels), such as the current screening suites based on tandem mass spectrometry, which allow testing for multiple conditions simultaneously. National and regional healthcare systems all around the world have been providing specific screening programs for infants that, due to the continuous technological process, comprise tests for an ever-increasing number of conditions [3].

As the number of conditions assessable by NBS is increasing, resulting in additional costs for the healthcare systems, it is necessary to identify solid criteria which can inform health decision-making. Economic evaluations (EEs) represent an increasingly popular tool for policy-makers to make informed and effective choices in healthcare resource allocation and guarantee the financial sustainability of healthcare systems [4]. By comparing “alternative courses of action in terms of both costs and consequences” [5], EEs allow evaluating the opportunity costs of a healthcare intervention versus the improvement resulting from the examined intervention [6].

Economic appraisal of healthcare technologies is a tool to maximize health gains and secure health outcomes in healthcare policy-making. In fact, some national governments have implemented strategies to use the evidence coming from EEs, mainly in reimbursement decisions [7]. Deciding on including new conditions in newborn screening programs, as well as making reimbursement choices on individual tests, requires a thoughtful assessment of the costs and effects of the screening and the identification of the most effective and efficient tests. In this sense, the results of EEs may provide guidance for recognizing and implementing (or de-implementing) technologies according to their impact on public health outcomes and the sustainability of healthcare systems. However, the use of economic evaluations to inform funding decisions on newborn screening programs seems limited [8,9,10].

Despite the similarity in screened conditions of many governments [3], to date, many discrepancies remain about the way NBS EEs are performed [11]. Conducting EEs of NBS presents several, unique challenges [11]. The first is the lack of data on long-term consequences. Follow-up information for the screened conditions is usually scarce or absent, therefore having to rely on model assumptions, with all the limitations that this entails. Moreover, accurate collection of costs to populate the analyses can be difficult, especially when a broader societal perspective is chosen and the number of parameters to consider extends beyond the direct cost of screening (e.g., spill-over effects on family members, long-term costs, etc.). In addition, estimating health outcome parameters in infants is problematic. Valuation techniques for pediatric health states have not been standardized yet and challenges arise because of the inability of children to evaluate their health states and the problems and biases resulting from adopting proxy respondents or using clinical outcomes [12,13].

The current state of the art of NBS EEs is unclear, as well as the most common methodological approaches used. Literature reviews on NBS have been conducted to synthesize evidence on cost-effectiveness of screening interventions for one or more conditions and to discuss specific methodological aspects in conducting economic evaluations of these screening programs [14,15,16,17]. To the best of our knowledge, a specific focus on the broad range of methodological aspects is still missing in the literature. Investigating these aspects may clarify methodological strengths and flaws, and provide practical insights to overcome the specific issues in conducting EEs of NBS. To achieve this goal, a scoping review of literature was conducted with the following research question: What is the methodological quality of economic evaluations (cost-effectiveness and cost–utility analyses) and what are the main challenges in performing these economic evaluations of newborn screening programs and technologies?

## 2. Materials and Methods

A scoping review was chosen as a research synthesis tool to identify relevant published EEs of NBS program. Scoping reviews can be used to examine how research is conducted on a certain topic or field, which is in line with our aim of studying the methodological quality of the economic evaluations and identifying the main challenges in performing economic evaluations [18]. The review followed the Preferred Reporting Items for Systematic reviews and Meta-Analyses (PRISMA) and its extension for Scoping Reviews (PRISMA-ScR) [19]. The search was conducted during 20–23 January 2020 on the following databases: MEDLINE (via PubMed), Embase, Web of Science, the Cochrane Library, and EMCare. Relevant articles were searched using different key strings which are indicative of NBS EEs (see Appendix A for full search filters). Moreover, the websites of the Health Technology Assessment (HTA) agencies belonging to the International Network of Agencies of Health Technology Assessment (INAHTA) were searched to retrieve additional reports and documents on EEs of NBS. The retrieved studies were screened by title and abstract according to the inclusion criteria. As CEAs and CUAs are the most popular tools for value-for-money recommendations and resource allocation in many countries [20], the screening was restricted to these kinds of EEs. Cost-effectiveness analyses (CEAs) and cost–utility analyses (CUAs) are two types of economic analyses comparing the costs of health interventions with an outcome expressed, respectively, in natural units (e.g., life-years saved) or quality-adjusted life years. In addition, only studies analyzing NBS in newborns up to one-month-old; published in English, Italian, or Dutch; and published in the last ten years were included in the review (a full list of inclusion and exclusion criteria is presented in Appendix B). A random sample of 10% of the identified records was screened by three researchers (PC, NB, and EVdA) in title/abstract and full-text screening to check for consistency. Disagreements were resolved through discussion. The remaining studies were screened by a single author (PC). Potential doubts were solved through an additional review by the aforementioned researchers. Subsequently, the following elements were charted: authors, year of publication, country of origin, methodological quality of the economic evaluation (see description below), and key findings related to the scoping review research question.

The quality of the studies identified after full-text screening was assessed using the guideline developed by Langer and colleagues in the form of a checklist [21]. This guideline, derived from existing tools to assess the quality of EEs, was designed to assess and improve the methodological quality of NBS economic studies. A synthesis of the categories and assessment items from this guideline is presented in Table 1. Specifically, a score of 0 ( = not addressed) or 1 ( = addressed) was assigned to each item of the 10 assessment categories for every EE. The score for each category was calculated as the average of the item scores belonging to that category (where relevant for every EE), with the aim of identifying the more and less represented aspects in NBS EE. A general score was then calculated per EE by averaging the category scores. In addition, the main methodological challenges encountered by the researchers and described in the studies were collected and analyzed using a qualitative synthesis approach (specifically, a thematic analysis process [22]). This process involved reading the EEs, identifying the common methodological issues and challenges reported by the researchers in developing their analyses, and categorizing the findings in common themes.

## 3. Results

### 3.1. Search Outcomes

The literature search identified 1203 records in five databases. After duplicate removals, 716 articles were considered eligible for screening by title and abstract. After full-text screening, 35 articles were included for the final assessment (see Appendix C for the PRISMA flow diagram) [23,24,25,26,27,28,29,30,31,32,33,34,35,36,37,38,39,40,41,42,43,44,45,46,47,48,49,50,51,52,53,54,55,56,57]. No additional studies were found on the websites of the INAHTA HTA agencies.

Most EEs came from the United States and Canada (*n* = 5 for each country), followed by France, Iran, the Netherlands, Spain, and the United Kingdom (*n* = 3 for each country), and various other countries (*n* = 12). The main assessed disease areas for NBS were congenital heart defects and metabolic disorders (*n* = 7 studies for each area), followed by severe combined immunodeficiency disorder and hearing loss (*n* = 5 for each of these diseases), cystic fibrosis and sickle cell disease (*n* = 4 for each of these), and others (*n* = 3).

### 3.2. Assessment Results

**Figure 1:**
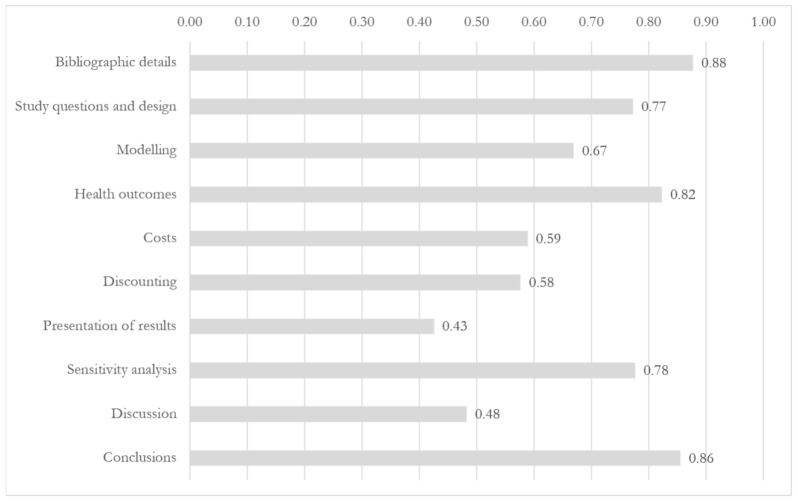
Category representation in the analyzed economic evaluations (score range: 0–1).

The results of the assessment based on the guideline are presented in Figure 1. “Bibliographic details”, “Conclusions”, and “Health Outcomes” were the most represented items, with overall scores of 0.88, 0.86, and 0.82, respectively. The lowest overall scores were associated to the categories “Discounting” (0.58), “Discussion” (0.48), and “Presentation of results” (0.43). This was mainly due to low scores reported within the categories, respectively, the items “Justification of discount rates”, “Generalizability and transferability of the economic results”, and “Present values and trends of costs and the population and payer level”. The overall characteristics of the EEs are available in Appendix D.

#### 3.2.1. Study Questions and Design

The main overlooked items in the category “Study questions and design” were “Setting” (clear description missing in 23 EEs, 66%), “Time horizon” (*n* = 12, 34%), and “Target population” (*n*= 8, 23%). The most frequent primary outcome measure was the Incremental Cost-Effectiveness Ratio (ICER) (*n*= 23, 61%), with two EEs not using a comparator and presenting the (Average) Cost-Effectiveness Ratio. Cost-effectiveness analysis (CEA) was the main type of EE (*n* = 27, 71%), while only five studies (13%) were presented as cost–utility analyses (CUA), using health outcomes adjusted by utility weights and time [31,38,43,50,57]. The great majority of the EE used cohort models to evaluate NBS (*n* = 32, 91%). Many of the studies (*n* = 20, 57%) were conducted from a healthcare perspective, while societal and third-payer perspective were, respectively, considered only in eight and three studies. The time horizon of the EEs, when relevant, ranged from 1 month to 100 years, and 10 (29%) of the included studies adopted a lifetime horizon. In terms of comparators, the majority of the retrieved studies explored the cost-effectiveness of a newborn screening introduced in a naïve setting (comparing a neonatal screening with absence of screening).

#### 3.2.2. Modeling

“Model validation” was reported in five EEs (14%), and this was mainly done by discussion with experts or comparison with previous studies [26,27,33,52,56]. A clear description of the assumptions made to build and populate the model was missing in eight EEs (23%). All EEs in this study were based on decision-analytic models, varying from quite basic projections based on observed (pilot) data to extensive models. Among the articles describing the model structure, decision trees were preferred to Markov models (*n* = 22 vs. 7). None of the EEs was a piggyback analysis (an EE study which is embedded in a clinical trial).

#### 3.2.3. Health Outcomes

The analysis of the retrieved EEs of NBS showed that the choice of outcomes was not consistent and uniform, as the measures were fairly distributed between natural units (*n* = 22, 63%) and quality-adjusted/daily-adjusted life years (QALYs/DALYs) (*n* = 18, 51%). A few EEs also considered health outcomes in monetary units (*n* = 7, 20%), in addition to natural units or QALYs/DALYs. One EE specifically reported health outcomes defined as intermediate (e.g., number of infants diagnosed preclinically or number of infant deaths because of the screened condition) [35].

#### 3.2.4. Costs

No EE in this scoping review considered all the relevant categories of costs recommended by Langer et al. [21], namely at the administrative level (cost of implementing, running, and evaluation of the screening) and at the individual patient level. In addition, 16 studies (46%) only addressed one of the two categories of costs. Program-related costs were absent in nearly 20% of the EEs; among these, administrative costs were the main overlooked category in the analysis. These include costs to set up a screening infrastructure, the costs to train the NBS personnel, and location and overhead costs. Many NBS EEs adopting a societal perspective also failed to include productivity losses; from this perspective, they can account for a large proportion of costs because illness, treatment, disability or death can affect the patients or the caretakers’ labour productivity or ability to work. For NBS, these costs include both lost parental wages [36] and potential losses incurred by the patients after they reach adulthood [54].

#### 3.2.5. Discounting

Discounting refers to the conversion of future costs and effects to their present value [5]. A discount rate for costs was included in 26 EEs (74%), while 20 studies (57%) also considered discounting health outcomes. Half of the retrieved EEs which discounted costs or outcomes did not justify the rationale behind the choice of a specific discounting rate.

#### 3.2.6. Presentation of Results

Most of the studies clearly presented absolute or incremental health outcomes and costs per newborn in their results. However, a small minority of the studies also presented values and trends of costs and health outcomes at the population level (*n* = 10, 29%) and only one EE differentiated these outputs by payer [23]. Coverage of screening was explicitly described in only one study [29], but coverage of universal screening newborn screening programmes may be close to 100% where screenings are easily accessible (e.g., free-of-charge screenings mandated as a public health service).

#### 3.2.7. Sensitivity Analysis

Sensitivity analysis is a technique that allows to quantify the sensitivity of the EE outcome due to the uncertainty of the information included in the EE [5]. It was conducted by the majority of authors in the retrieved EEs: 30 (86%) of the studies included a specific reference to deterministic and/or probabilistic sensitivity analysis. Results of the analyses were extensively reported and discussed in most of the studies (*n* = 27, 77%). In their EE on a panel of screening for metabolic disorders, Thiboonboon et al. included a budget impact analysis to estimate resource impact over a 10-year interval [30]. An expected value of perfect information analysis was additionally undertaken by Bessey et al. [44], who calculated the value of eliminating all uncertainty in the model parameters.

#### 3.2.8. Discussion

Overall, the assessed EEs lacked a clear and detailed descriptions of the generalizability of their results and the extent to which they were applicable to other settings. Ethical and transferability issues were also constantly overlooked: less than one-third of the EEs (29%) considered the issues concerning the dissemination of the assessed screening intervention.

#### 3.2.9. Conclusions

Nearly all studies (*n* = 31, 89%) presented conclusions which resulted directly from the results of the EE. Only four papers lack a clear answer to the research question presented as the objective of the study [29,52,54,56].

### 3.3. Qualitative Assessment

The qualitative assessment of the studies allowed us to identify the most common themes on methodological issues as presented and discussed by the authors of the retrieved NBS EEs. They are presented in the sections below.

#### 3.3.1. Study Questions and Design

Scientific research and technological advancements have increased the number of comparators in the years, and many options may already be in place to screen for a neonatal preventable condition. Some authors suggested that an evidence-based prioritization can justify the focus on a limited number of alternatives [30], while others recommended an incremental approach by expanding the number of interventions to compare, although, for the latter, the information requirement on the intervention parameters is more stringent [54]. As for population, only a limited number of EEs considered a targeted screening for subgroups of newborns, probably because of growing consensus that ethnically targeted neonatal screening is not an acceptable public health strategy because of ethical reasons [58].

The time horizon adopted in the identified EEs was heterogeneous and the reason behind the time horizon choice was not always stated. Not being able to adopt a lifetime horizon was often listed as a limitation of the study [26,34,41,57]. On the other hand, Gantt et al. recognized that a prolonged time horizon in some disease settings can “dramatically shift” the results of the study, but it would pose particular issues when the condition is rare, the costs of care are high, or designing a clinical study to screen patients with the underlying condition is ethically difficult [36]. An explanation for a shorter time horizon is also present in [27,34]: researchers recognized that extending the time horizon over the “pediatric population” time span would have required data on costs and health outcomes for adult populations. For this reason, for example, while investigating the cost-effectiveness of a NBS for severe combined immunodeficiency, Ding et al. used two different time horizons: five years for assessing health outcomes and lifetime for assessing survival [35].

#### 3.3.2. Modeling

The most common models in EEs are decision trees, which represent a formal structure of the decisions and chance events in the order in which they occur, and Markov models, which include events as transitions from one health state to another over time [59]. In [25], the authors mentioned that a Markov model was not used because of the limited time horizon of the analysis, while Van der Ploeg et al. [49] considered a Markov model to be unnecessary, since health outcomes for the screened condition could be adequately represented in a deterministic modeling approach. Overall, however, the choice for a modeling approach was not always clearly substantiated by the researchers. The only exception appeared to be an EE on sickle-cell disease screening [34]: this EE adopted a discrete-event simulation modeling approach because it was considered particularly apt to represent the risk and interdependencies among disease complications, even though this approach required the collection of more data.

#### 3.3.3. Health Outcomes

Up to now, there is no consensus on what an appropriate health outcome measure should be in EEs of NBS. Langer et al. recognized that the relevance for the patient should drive the choice of the outcome measure [21]. However, the decision made by the researchers in the identified NBS EEs demonstrated that the choices had often been driven by incomplete or low-quality data, which resulted in analysis limitations. Some researchers explicitly stated that QALYs would have been a preferred measure, but insufficient parameters on the quality of life led to the use of LYs gained [32]. Some concerns associated with choosing a specific health measure were related to the loss of informative aspects on potential health benefits: for example, in the study by Tobe et al., the choice of “DALYs averted” as the main outcome measure did not allow incorporating information related to other potential health benefits, such as morbidities avoided in the long term when quality weights cannot be collected [42]; the use of the number of correctly detected cases of hearing loss was preferred over the outcome measures on language and speech development in children, which might have been more informative, in [41]; and Hatam et al. did not include the spill-over effects on patient’s families [55], even though this could have increased the health benefits of executing the investigated NBS program. Overall, the exclusion of health outcomes was mainly driven by insufficient information and substantial uncertainty on long-term outcomes [36]. Many studies used retrospective data, systematic reviews, or expert opinions while populating the model, potentially resulting in subjective choices in the analyses and hence affecting the generalizability of the results.

#### 3.3.4. Costs

Specific costs associated to NBS for genetic diseases were not included in the assessed studies, but highlighted by the researchers as relevant for the interpretation of the results: the cost of genetic counselling for parents and siblings to investigate a carrier status [32,34]; the “emotional costs” associated with a false positive result or a heterozygous diagnosis in the newborn; and the costs of change in reproductive decisions following a positive diagnosis [32]. Overall, researchers seemed to rely on workarounds or proxy measures to include some costs in their analyses, especially in the studies adopting a societal perspective. In some EEs, screening costs were estimated using a micro-costing approach. More specifically, they used a “time-and-motion” approach to derive cost estimates (tracking the time to perform the test and multiplying it by the value of average hourly compensation of the personnel involved) [39,47,49,53,56].

#### 3.3.5. Discounting

The choice of a discounting rate is linked with the time horizon adopted in the EE, as well as the national HTA guidelines used to inform the study. Many studies which adopted a discounting rate for health outcomes and/or costs did not state or justify the rationale of the rates; in contrast, an explicit justification for their absence was present in all studies which did not use a discount rate (because of a time horizon shorter than one year).

#### 3.3.6. Sensitivity Analysis

All authors including sensitivity analyses in their studies specifically stated that this was to account for the uncertainties of the parameters included in the model. In addition to this reason, Tobe et al. used sensitivity analysis to accommodate for the geographical and socioeconomic diversity within a target country [42]. Several EEs also used additional methods to explore other types of uncertainty. Some studies presented the results under different model structure/input assumptions (for instance, sensitivity and specificity of the screening test) through different scenario analyses [24,36,38,40,47,56].

#### 3.3.7. Discussion

The authors of the few studies presenting generalizability and transferability issues considered different elements to address these items. While most of the researchers considered the difference in population and health care systems as the main obstacle to generalizability [24,26,28,52,56], they also used sensitivity analysis to enhance the generalizability of the results [24,52] and called for further research to increase their transferability [26,27,30,42]. Distributional issues mainly considered the limited access to service (geographically and/or logistically) as the “bottleneck” for scaling-up the investigated NBS intervention [50]. Ewet et al. finally considered the “ethical dilemma” of setting up new randomized clinical studies, which would require the blinding of medical staff to investigate the added value of incorporating a new screening intervention [33].

## 4. Discussion

The goal of this scoping review was to analyze the methodological quality of NBS EEs and collect evidence on the main challenges of conducting this category of studies. The analysis of 38 EEs through the framework of a specific NBS guideline showed that the methodological quality of these NBS papers was heterogenous. Some items of the guideline were constantly underreported in the studies (e.g., generalizability and transferability of results, justification of discount rates, time trends of costs at the population and payer levels). In addition, the qualitative information contained in the identified EEs highlighted the most common challenges in this field and the resulting decisions or approaches taken by the authors around common themes of EEs, including health outcomes, costs and modeling decisions.

As for the choice of the outcome measure, the review revealed a high level of heterogeneity among EEs. Health benefits, expressed as natural units (for example, number of cases detected or healthy life years gained with a screening program), may be easy to quantify, but they may limit the comparability with other interventions/screening. For this reason, the use of QALY has become a recognized way to allow for comparability between interventions for various disorders, by standardizing mortality, morbidity and health status in a single measure [60]. Several reimbursement agencies now require the use of QALY when assessing health interventions, which has increased the number of published cost–utility studies [61]. However, the use of QALYs requires knowledge of utility measures for the target population (in this case, newborns), and many authors highlighted the challenge in collecting and interpreting pediatric utility weights.

A review by Grange et al. in 2007 identified 16 generic health-related quality of life (HRQoL) measures for children less than five years of age, but none of them was conceptually and psychometrically robust [62] At the present day, instruments to measure utility in children are still scarce, and a valid general measure that can help in assessing utility in young children does not yet exist [63]. The Health Utility Index (HUI), a validated health status index, is considered to be valid in children from five years old onwards, but evidence on its reliability in children under five is still lacking [64]. In addition, the Euro-Quality of Life 5-Dimension questionnaire on QoL (EQ-5D) is available in a pediatric version, but only targeting children aged 7–12 years [65]. Finally, a proxy version of The Child Health Utility 9D, a pediatric HRQoL measure developed for children aged 7–17, is currently being trialed for the under five age group [66]. Future research will need to focus on the development of a generic instrument specifically designed for the very young age group, in order to harmonize EEs in this population and increase comparability of results across interventions [67]. In addition, identifying and quantifying appropriate outcome measures in adults is important when NBS assess non-treatable conditions, to take into account the quality of life loss due to the earlier knowledge of the non-treatability status from the parents’ perspective and the potential influence on their future reproductive choices.

Because of their ability to intercept presymptomatic diseases, screening programmes can lead to savings, and this is particularly true when a treatable condition is identified early at the beginning of life [68]. This reason makes it important to consider all the relevant costs in NBS EEs. Nevertheless, none of the analyzed EEs considered all relevant costs related to a NBS intervention, showing that accounting for all relevant costs is a problematic aspect for EE in NBS field. New methodological research in economics of child healthcare can provide further guidance in determining an appropriate research framework for cost inputs (for example, guidelines such as the one adopted in this study or generalized models for cost-effectiveness appraisal [69]).

In terms of comparators, the cost-effectiveness of an assessed screening is intrinsically influenced by the choice of the comparator screening program. In EEs, the gold standard comparator should reflect the most relevant options as used in clinical practice. In newborn screening, this can often be represented by the “no screening” option. Nevertheless, the routine clinical practice for screening can consist of already existent screening programs or technologies. Increasing the number of comparators can increase the informativity of the EE, but it requires data on the available interventions and can further complicate the analysis. The qualitative analysis of the identified studies proved this point: the authors often limited the number of comparators and prioritized their choice based on the amount of available information on alternative routine interventions (including the “no screening” option). A high-quality and transparent model should include a proper presentation of the assessed comparators and a justification of their choice (national guidelines, lack of data, etc), as indeed remarked by the European Network for Health Technology Assessment in its methodological guidance [70]. In addition, more research initiatives should be set to collect data on available NBS to allow researchers to take into account the appropriate comparators in future EEs.

One of the main challenges in EEs is setting an appropriate time horizon for the analysis. The choice of a time horizon affects the final measure (e.g., ICER) in a cost-effectiveness or cost–utility analysis [71]. The selection of a time horizon depends on the scope of the analysis and the nature of intervention [5,72]. As NBS can prevent death and/or minimize morbidity (chronic conditions or disabilities), longer time horizons can reflect the relevant long-term cost and health consequences related to the early interventions enabled by NBS programs. However, relatively short time horizons (e.g., one year) adopted by the majority of the studies might be due to the difficulties researchers faced in the collection of long-term evidence to populate the models. The collection of more long-term data on diseases that can be detected by NBS could also help researchers to incorporate longer time horizons.

This review confirmed the importance of decision models also in NBS EEs. Decision modeling uses mathematical relationships between parameters to describe a series of outcomes coming from possible scenarios involving healthcare interventions [73]. Building good models, however, requires high-quality evidence and can be time- and resource-consuming. The discrepancy between the choice of decision trees and Markov models in this review might be related with data unavailability on the long-term consequences of NBS/underlying condition, and therefore hinder the construction of more data-hungry models (such as Markov models with multiple health states, which would necessitate calculation of transition probabilities).

Finally, the data used to develop decision-analytic models for EEs of NBS can often lead to uncertainty. This may be related to many reasons, including the rarity of the conditions assessed by the screening and the long-time horizons used in the model. By looking at the EEs in this review, it was clear that researchers usually handled these challenges by the employment of assumptions or proxy measures (e.g., opinions by experts). However, this comes at a cost for the accuracy/precision of the results and conclusions of the EE. Sensitivity analysis has a potential to comprehensively address uncertainty [74] and guide researchers in the choice of the input parameters to prioritize in their research. However, it does not compensate for the need of more research on input parameters, in order to reduce the confidence interval resulting from these studies and identify the optimal cost-effective approach in NBS screening [75].

This study presents some limitations. First, only cost-effectiveness and cost–utility analyses developed in the last ten years were selected, for feasibility purposes. The temporal limit of the search and the exclusion of cost–benefit and cost-minimization analyses may have influenced the results of this review, as well as the inclusion of economic evaluations only written in Dutch, English, or Italian. Second, we used the only available appraisal tool for NBS EEs in the literature, which is not validated. This influenced the study results, as other generic available tools may provide different ways to assess the quality of NBS EEs [76]. Furthermore, Langer’s tool for NBS EEs is presented as a checklist and our quantitative assessment assigned the same value to each item for converting it to a quality score. Future research could develop a tailored version of these guidelines by weighing the categories and their sub-items and by setting a cut-off to define good quality economic evaluations. However, the guideline by Langer et al., although not validated, was specifically developed for EEs of neonatal screenings, and assumed to be most consistent to assess the quality of NBS EEs in the form of a checklist. Finally, and possibly more importantly, the adopted guideline allowed identifying whether an EE was adequately performed, which is not the same as identifying methodological challenges. The qualitative approach allowed us to synthesize the main issues and limitations in the studies in order to complement this study’s research question. However, this was limited to the information disclosed by the authors (which, in turn, may have been narrowed by the word limit requirements of most scientific journals).

## 5. Conclusions

This study aimed at closing the existing gap on methodological quality of EEs and the challenges associated with them. It identified items which are frequently overlooked by researchers; described the challenges around common themes such as the choice of outcomes, costs, and comparators in NBS EEs; and considered the methodological choices made to address them. More research is needed to systematically investigate the main strategies by HTA researchers and intercept the common trends in this field of EEs. The results of this scoping review can lay the groundwork for future projects investigating, among others, the development of a generic HRQoL instrument designed for newborns, appropriate outcomes measures when the condition is not treatable, a new framework for a clear definition of cost inputs, and a transparent model for prioritization of EE comparators. In addition, this review can possibly aid researchers to develop a methodological guidance to harmonize the process in performing NBS EEs, improve their transparency, and increase their transferability, so that policy-makers can rely on high-quality research to inform policy decision on neonatal screening interventions.

## Figures and Tables

**Table 1:** A synthesis of the assessment categories from the guidelines to assess the quality of NBS economic evaluations (by Langer et al. [21]).

Category	Items	Score (0/1)
Bibliographic details	Authors Institutional affiliation of authors Source of funding Title Source of publication Publication type	S1 S2 S3 S4 S5 S6
	Average score for “Bibliographic details”	Avg. S1–S6
Study question and design	Study question Intervention Control Target population Time horizon Setting Perspective Study design Type of economic evaluation Study population Primary outcome measure	S7 S8 S9 S10 S11 S12 S13 S14 S15 S16 S17
	Average score for “Study question and design”	Avg. S7–S17
Modeling	Model type Model structure Model assumptions Sources used to develop and/or populate the model Cycle length Health states and transitions Model validation	S18 S19 S20 S21 S22 S23 S24
	Average score for “Modeling”	Avg. S18–S24
Health outcomes	Health outcomes measured in natural units Health outcomes adjusted by utility weights or health state preference scores Health outcomes measured in monetary units Intermediate health outcomes Non-health outcomes	S25 S26 S27 S28 S29
	Average score for “Health outcomes”	Avg. S25–S29
Costs	Patient-related costs Programme-related costs	S30 S31
	Average score for “Costs”	Avg. S30–S31
Discounting	Discount rate for costs Discount rate for health outcomes Justification of discount rates	S32 S33 S34
	Average score for “Discounting”	Avg. S32–S34
Presentation of results	Absolute and incremental health outcomes per newborn Absolute and incremental costs per newborn ICER for the primary outcome measure Present values and trends of costs and health outcomes at the population level Present values and trends of costs at the population level differentiated by payer Coverage of screening	S35 S36 S37 S38 S39 S40
	Average score for “Presentation of results”	Avg. S35–S40
Sensitivity analysis	Parameter uncertainty Modeling uncertainty Methods of sensitivity analysis Results of sensitivity analysis	S41 S42 S43 S44
	Average score for “Sensitivity analysis”	Avg. S41–S44
Discussion	Limitations of the study Generalizability and transferability of the economic evaluation results	S45 S46
	Average score for “Discussion”	Avg. S45–S46
Conclusions	Validity of conclusions with regard to the results of the economic evaluation Validity of conclusions with regard to the objective of the economic evaluation	S47 S48
	Average score for “Conclusions”	Avg. S47–S48

**Figure A1:**
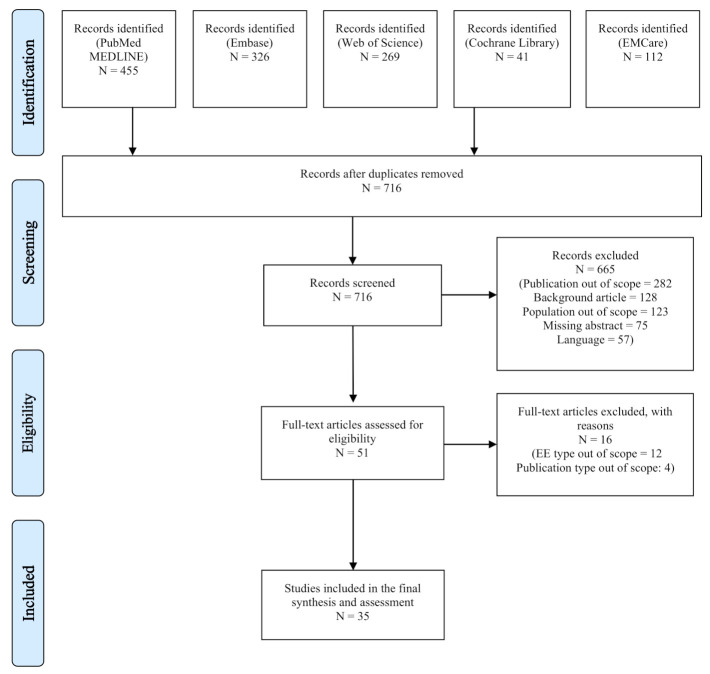
PRISMA Flow Diagram.

## Abbreviations

ACER	Average Cost-Effectiveness Ratio
CBA	Cost–Benefit Analysis
CEA	Cost-Effectiveness Analysis
CUA	Cost–Utility Analysis
DALY	Daily-Adjusted Life Year
EE	Economic Evaluation
HRQoL	Health-Related Quality of Life
HTA	Health Technology Assessment
ICER	Incremental Cost-Effectiveness Ratio
INAHTA	International Network of Agencies of Health Technology Assessment
LY	Life Year
NBS	Newborn screening
PRISMA	Preferred Reporting Items for Systematic Reviews and Meta-Analyses
QALD	Quality-Adjusted Life Day
QALM	Quality-Adjusted Life Months
QALY	Quality-Adjusted Life Year

## Appendix A. Search Filters

### Appendix A.1. PubMed

(((("economic evaluations"[tw] OR "economic evaluation"[tw] OR "Cost-Benefit Analysis"[Mesh] OR "Cost-Benefit Analysis"[tw] OR "Benefits and Costs"[tw] OR "Cost Benefit"[tw] OR "Cost Benefit Analysis"[tw] OR "Cost Benefit Data"[tw] OR "Cost Effectiveness"[tw] OR "Cost Effectiveness Analysis"[tw] OR "Cost Effective"[tw] OR "Cost Utility"[tw] OR "Cost Utility Analysis"[tw] OR "Costs and Benefits"[tw] OR "Marginal Analysis"[tw]) AND ("Neonatal Screening"[majr] OR "Neonatal Screening"[ti] OR "Neonatal Screenings"[ti] OR "newborn screening"[ti] OR "newborn screenings"[ti] OR "Newborn Infant Screening"[ti] OR (("screening"[ti] OR "screenings"[ti] OR "screen"[ti] OR "screened"[ti] OR screen*[ti]) AND ("Infant, Newborn"[majr] OR newborn*[ti] OR neonat*[ti])))) OR (("economic evaluations"[ti] OR "economic evaluation"[ti] OR "Cost-Benefit Analysis"[majr] OR "Cost-Benefit Analysis"[ti] OR "Benefits and Costs"[ti] OR "Cost Benefit"[ti] OR "Cost Benefit Analysis"[ti] OR "Cost Benefit Data"[ti] OR "Cost Effectiveness"[ti] OR "Cost Effectiveness Analysis"[ti] OR "Cost Effective"[ti] OR "Cost Utility"[ti] OR "Cost Utility Analysis"[ti] OR "Costs and Benefits"[ti] OR "Marginal Analysis"[ti]) AND ("Neonatal Screening"[Mesh] OR "Neonatal Screening"[tw] OR "Neonatal Screenings"[tw] OR "newborn screening"[tw] OR "newborn screenings"[tw] OR "Newborn Infant Screening"[tw] OR (("screening"[tw] OR "screenings"[tw] OR "screen"[tw] OR "screened"[tw] OR screen*[tw]) AND ("Infant, Newborn"[Mesh] OR newborn*[tw] OR neonat*[tw])))) OR "Neonatal Screening/economics"[majr]) AND ("1 January 2010"[PDAT] : "31 December 2020"[PDAT]))

### Appendix A.2. Embase

((((exp *”economic evaluation"/OR "economic evaluations".ti,ab OR "economic evaluation".ti,ab OR "Cost-Benefit Analysis".ti,ab OR "Benefits and Costs".ti,ab OR "Cost Benefit".ti,ab OR "Cost Benefit Analysis".ti,ab OR "Cost Benefit Data".ti,ab OR "Cost Effectiveness".ti,ab OR "Cost Effectiveness Analysis".ti,ab OR "Cost Effective".ti,ab OR "Cost Utility".ti,ab OR "Cost Utility Analysis".ti,ab OR "Costs and Benefits".ti,ab OR "Marginal Analysis".ti,ab) AND (*"Newborn Screening"/OR "Neonatal Screening".ti OR "Neonatal Screenings".ti OR "newborn screening".ti OR "newborn screenings".ti OR "Newborn Infant Screening".ti OR (("screening".ti OR "screenings".ti OR "screen".ti OR "screened".ti OR screen*.ti) AND (*"Newborn"/OR newborn*.ti OR neonat*.ti)))) OR ((exp *"economic evaluation"/OR "economic evaluations".ti OR "economic evaluation".ti OR "Cost-Benefit Analysis".ti OR "Benefits and Costs".ti OR "Cost Benefit".ti OR "Cost Benefit Analysis".ti OR "Cost Benefit Data".ti OR "Cost Effectiveness".ti OR "Cost Effectiveness Analysis".ti OR "Cost Effective".ti OR "Cost Utility".ti OR "Cost Utility Analysis".ti OR "Costs and Benefits".ti OR "Marginal Analysis".ti) AND (*"Neonatal Screening"/OR "Neonatal Screening".ti,ab OR "Neonatal Screenings".ti,ab OR "newborn screening".ti,ab OR "newborn screenings".ti,ab OR "Newborn Infant Screening".ti,ab OR (("screening".ti,ab OR "screenings".ti,ab OR "screen".ti,ab OR "screened".ti,ab OR screen*.ti,ab) AND (*"Newborn"/OR newborn*.ti,ab OR neonat*.ti,ab))))) AND (2010 OR 2011 OR 2012 OR 2013 OR 2014 OR 2015 OR 2016 OR 2017 OR 2018 OR 2019 OR 2020).yr) NOT (conference review or conference abstract).pt

### Appendix A.3. Web of Science

((ts = ("economic evaluation" OR "economic evaluations" OR "economic evaluation" OR "Cost-Benefit Analysis" OR "Benefits and Costs" OR "Cost Benefit" OR "Cost Benefit Analysis" OR "Cost Benefit Data" OR "Cost Effectiveness" OR "Cost Effectiveness Analysis" OR "Cost Effective" OR "Cost Utility" OR "Cost Utility Analysis" OR "Costs and Benefits" OR "Marginal Analysis") AND ti = (*"Newborn Screening" OR "Neonatal Screening" OR "Neonatal Screenings" OR "newborn screening" OR "newborn screenings" OR "Newborn Infant Screening" OR (("screening" OR "screenings" OR "screen" OR "screened" OR screen*) AND ("Newborn" OR newborn* OR neonat*)))) OR (ti = ("economic evaluation" OR "economic evaluations" OR "economic evaluation" OR "Cost-Benefit Analysis" OR "Benefits and Costs" OR "Cost Benefit" OR "Cost Benefit Analysis" OR "Cost Benefit Data" OR "Cost Effectiveness" OR "Cost Effectiveness Analysis" OR "Cost Effective" OR "Cost Utility" OR "Cost Utility Analysis" OR "Costs and Benefits" OR "Marginal Analysis") AND ts = ("Neonatal Screening" OR "Neonatal Screening" OR "Neonatal Screenings" OR "newborn screening" OR "newborn screenings" OR "Newborn Infant Screening" OR (("screening" OR "screenings" OR "screen" OR "screened" OR screen*) AND ("Newborn" OR newborn* OR neonat*))))) AND py = (2010 OR 2011 OR 2012 OR 2013 OR 2014 OR 2015 OR 2016 OR 2017 OR 2018 OR 2019 OR 2020) NOT dt = (meeting abstract)

### Appendix A.4. Cochrane

(("economic evaluation" OR "economic evaluations" OR "economic evaluation" OR "Cost-Benefit Analysis" OR "Benefits and Costs" OR "Cost Benefit" OR "Cost Benefit Analysis" OR "Cost Benefit Data" OR "Cost Effectiveness" OR "Cost Effectiveness Analysis" OR "Cost Effective" OR "Cost Utility" OR "Cost Utility Analysis" OR "Costs and Benefits" OR "Marginal Analysis") AND (*"Newborn Screening" OR "Neonatal Screening" OR "Neonatal Screenings" OR "newborn screening" OR "newborn screenings" OR "Newborn Infant Screening" OR (("screening" OR "screenings" OR "screen" OR "screened" OR screen*) AND ("Newborn" OR newborn* OR neonat*)))):ti,ab,kw NOT (conference abstract):pt AND py = (2010 OR 2011 OR 2012 OR 2013 OR 2014 OR 2015 OR 2016 OR 2017 OR 2018 OR 2019 OR 2020) NOT dt = (meeting abstract)

### Appendix A.5. EMCare

((((exp *"economic evaluation"/OR "economic evaluations".ti,ab OR "economic evaluation".ti,ab OR "Cost-Benefit Analysis".ti,ab OR "Benefits and Costs".ti,ab OR "Cost Benefit".ti,ab OR "Cost Benefit Analysis".ti,ab OR "Cost Benefit Data".ti,ab OR "Cost Effectiveness".ti,ab OR "Cost Effectiveness Analysis".ti,ab OR "Cost Effective".ti,ab OR "Cost Utility".ti,ab OR "Cost Utility Analysis".ti,ab OR "Costs and Benefits".ti,ab OR "Marginal Analysis".ti,ab) AND (*"Newborn Screening"/OR "Neonatal Screening".ti OR "Neonatal Screenings".ti OR "newborn screening".ti OR "newborn screenings".ti OR "Newborn Infant Screening".ti OR (("screening".ti OR "screenings".ti OR "screen".ti OR "screened".ti OR screen*.ti) AND (*"Newborn"/OR newborn*.ti OR neonat*.ti)))) OR ((exp *"economic evaluation"/OR "economic evaluations".ti OR "economic evaluation".ti OR "Cost-Benefit Analysis".ti OR "Benefits and Costs".ti OR "Cost Benefit".ti OR "Cost Benefit Analysis".ti OR "Cost Benefit Data".ti OR "Cost Effectiveness".ti OR "Cost Effectiveness Analysis".ti OR "Cost Effective".ti OR "Cost Utility".ti OR "Cost Utility Analysis".ti OR "Costs and Benefits".ti OR "Marginal Analysis".ti) AND (*"Neonatal Screening"/OR "Neonatal Screening".ti,ab OR "Neonatal Screenings".ti,ab OR "newborn screening".ti,ab OR "newborn screenings".ti,ab OR "Newborn Infant Screening".ti,ab OR (("screening".ti,ab OR "screenings".ti,ab OR "screen".ti,ab OR "screened".ti,ab OR screen*.ti,ab) AND (*"Newborn"/OR newborn*.ti,ab OR neonat*.ti,ab))))) AND (2010 OR 2011 OR 2012 OR 2013 OR 2014 OR 2015 OR 2016 OR 2017 OR 2018 OR 2019 OR 2020).yr)

## Appendix B. Eligibility Criteria

### Appendix B.1. Inclusion Criteria

- Full economic evaluations (CEAs, CUAs) of NBS programs and technologies
- Studies focusing on newborns (up to one-month-old)
- English, Italian, Dutch language
- Publication date: from 1 January 2010

### Appendix B.2. Exclusion Criteria

- Partial economic evaluations of NBS or cost–benefit/cost-minimization analyses
- Not economic evaluation studies
- Reviews/qualitative studies
- Studies without available full-text

## Appendix D. Overall Results

**Table A1:** Descriptive analysis of the main assessment items from the economic evaluations.

N.	First Author	Year	Country	Topic of the Screening Intervention	Primary Outcome Measure	Type of Economic Evaluation	Perspective	Study Design	Time Horizon	Study Population	Control	Model Structure	Cycle Length (Markov Model)	Health Outcomes in Natural Units	Health Outcomes Adjusted in Utility Weights	Discount Rates	Sensitivity Analysis	Overall Score
22	Sicuri	2012	Spain	Chagas disease	ICER	CUA	Healthcare	Not clear/Not described	Not defined/Not clear	All newborns	No screening	Decision tree			QALYs gained	3% (costs)	Probabilistic	0.71
23	Tobe	2013	China	Hearing impairment	ACER	CEA	Healthcare	Cohort-based study	Not defined/Not clear	All newborns	No screening	Decision tree			DALYs averted	No discount rate	Deterministic	0.82
24	Malec	2014	US	Intracranial hemorrhage	ICER	CUA	Societal	Cohort-based study	1 month	Targeted-screening newborns	Not clear/Not described	Decision tree			QALDs gained	No discount rate	Deterministic	0.63
25	Nshimyumukiza	2014	Canada	Cystic fibrosis	Cost per case detected	CEA	Healthcare	Cohort-based study	5 years	All newborns	No screening	Markov model	1 year	Case detected		3% (costs)	Deterministic	0.76
26	Schreiber	2014	Canada	Biliary atresia	ICER	CEA	Healthcare	Cohort-based study	10 years	All newborns	No screening	Decision tree, Markov model	1 year	Life-years gained		5% (costs)	Not clear/Not described	0.68
27	McGann	2015	Angola	Sicke cell anemia	Cost per healthy life year gained	CEA	Not clear/Not described	Individual patient study	Not defined/Not clear	All newborns	Not clear/Not described	Not clear/Not relevant		Life-years gained		3% (costs), 3% (health outcomes)	Not clear/Not described	0.64
28	Mogul	2015	US	Biliary atresia	ICER	CEA	Societal	Cohort-based study	20 years	Not clear/Not described	No screening	Markov model	1 year	Life-years gained		3% (costs)	Deterministic, Probabilistic	0.66
29	Thiboonboon	2015	Thailand	Metabolic disorders	ICER	CUA	Societal	Cohort-based study	100 years	Targeted-screening newborns	Current practice	Decision tree, Markov model	1 year	Life-years gained	QALYs gained	3% (costs), 3% (health outcomes)	Deterministic, Probabilistic	0.9
30	Vallejo-Torres	2015	Spain	Biotinidase deficiency	ICER	CEA *	Healthcare	Cohort-based study	Lifetime	All newborns	No screening	Decision tree			QALYs gained	3% (costs), 3% (health outcomes)	None	0.86
31	van der Ploeg	2015	The Netherlands	Cystic fibrosis	CER	CEA	Societal	Cohort-based study	Lifetime	All newborns	No screening	Not clear/Not relevant		Case detected		3% (costs), 3% (health outcomes)	Deterministic	0.85
32	Ewer	2012	UK	Congenital heart defects	Not defined/Not clear	CEA	Healthcare	Cohort-based study	Not defined/Not clear	All newborns	Clinical detection	Decision tree				3.5% (costs), 3.5% (health outcomes)	Deterministic, Probabilistic	0.84
33	Castilla-Rodríguez	2016	Spain	Sicke-cell disease	ICER	CEA	Healthcare	Cohort-based study	10 years	All newborns	Clinical detection	Not clear/Not relevant		Life-years gained	QALYs gained	3% (costs), 3% (health outcomes)	Deterministic	0.84
34	Ding	2016	US	Sever combined immunodeficiency syndrome	ICER	CEA, CBA	Not clear/Not described	Not clear/Not described	5 years for outcome, lifetime for survival	All newborns	No screening	Not clear/Not relevant		Life-years gained		3% (costs), 3% (health outcomes)	Deterministic, Probabilistic	0.66
35	Gantt	2016	US	Congential cytomegalovirus infection	Not defined/Not clear	CEA	Not clear/Not described	Not clear/Not described	Not defined/Not clear	Targeted-screening newborns	No screening	Not clear/Not relevant		Deaths averted		1% (costs)	Not clear/Not described	0.6
36	Hatam	2016	Iran	Metabolic disorders	ICER	CUA	Societal	Cohort-based study	Not defined/Not clear	All newborns	No screening	Decision tree			QALYs gained	3% (costs), 3% (health outcomes)	Deterministic	0.73
37	Kuznik	2016	Sub-Saharan Africa (47 countries)	Sicke cell disease	ICER	CEA(*)	Healthcare	Cohort-based study	Lifetime	All newborns	No screening	Markov model	1 year		DALYs averted	No discount rate	Deterministic, Probabilistic	0.78
38	Seror	2016	France	Cystic fibrosis	Not defined/Not clear	CEA	Healthcare	Cohort-based study	Not defined/Not clear	All newborns	Current practice	Not clear/Not relevant		Case detected		3% (costs)	None	0.58
39	Chiou	2017	Taiwan	Hearing impariment	ICER	CUA	Not clear/Not described	Cohort-based study	Not defined/Not clear	All newborns	No screening	Decision tree, Markov model	1 year		QALYs gained	3% (costs), 3% (health outcomes)	Deterministic, Probabilistic	0.73
40	Heidari	2017	Iran	Hearing impairment	Not clear	CEA	Healthcare	Cohort-based study	1 year	All newborns	Current practice	Decision tree		Case detected		No discount rate	Deterministic	0.65
41	Tobe	2017	China	Congenital heart defects	Not defined/Not clear	CEA	Societal	Cohort-based study	Lifetime	All newborns	Current practice	Decision tree		Case detected	DALYs averted	3% (costs)	Deterministic	0.84
42	Hamers	2012	France	acyl-CoA dehydrogenase deficiency	ICER	CEA *	Societal	Cohort-based study	Lifetime	All newborns	No screening	Decision tree		Life-years gained, deatsh averted	QALYs gained	4% (costs), 4% (health outcomes)	Deterministic, Scenario Analysis	0.68
43	Bessey	2018	UK	Adrenoleuco- dystrophy	ICER	CEA	Healthcare	Cohort-based study	Lifetime	All newborns	No screening	Decision tree		Case detected	QALYs gained	3.5% (costs), 3.5% (health outcomes)	Deterministic, Probabilistic	0.78
44	Bessey	2019	UK	Severe combined immunodeficiency	ICER	CEA	Healthcare	Cohort-based study	Not defined/Not clear	All newborns	No screening	Decision tree			QALYs gained	3.5% (costs), 1.5% (health outcomes)	Deterministic, EVPI	0.81
45	Binquet	2019	France	Congenital toxoplasmosis	Cost per outcome avoided	CEA	Healthcare	Cohort-based study	1/15 years	All newborns	Current practice	Decision tree		Case detected		3% (costs), 3% (health outcomes)	Deterministic	0.78
46	Trujillo	2019	Colombia	Congenital heart defects	ICER	CEA	Societal	Cohort-based study	Not defined/Not clear	Not clear/Not described	Clinical detection	Decision tree		Case detected		No discount rate	Deterministic, Probabilistic	0.82
47	Narayen	2019	The Netherlands	Congenital heart defects	Cost per case detected	CEA	Healthcare	Cohort-based study	Not defined/Not clear	All newborns	No screening	Decision tree		Case detected		No discount rate	Deterministic	0.78
48	van der Ploeg	2019	The Netherlands	Severe combined immunodeficiency syndrome	ICER	CEA	Healthcare	Cohort-based study	Lifetime	All newborns	No screening	Not clear/Not relevant		Deaths averted	QALYs gained	4% (costs), 1.5% (health outcomes)	Deterministic	0.74
49	Fox	2020	Canada	Congenital adrenal hyperplasia	ICER	CEA *	3rd payer	Cohort-based study	Not defined/Not clear	All newborns	No screening	Decision tree				No discount rate	Deterministic, Probabilistic	0.71
50	Mukerji	2020	Canada	Congenital heart defects	ICER	CEA	3rd payer	Cohort-based study	Lifetime	All newborns	No screening	Markov model	1 month		QALMs gained	1.5% (costs), 1.5% (health outcomes)	Deterministic, Probabilistic	0.72
51	Langer	2012	Germany	Hearing impairment	ICER	CEA	Healthcare	Cohort-based study	Lifetime	All newborns	No screening	Decision tree		Case detected		No discount rate	Deterministic, Probabilistic	0.79
52	Roberts	2012	UK	Congenital heart defects	ICER	CEA	Healthcare	Cohort-based study	1 year	All newborns	Clinical detection	Decision tree		Case detected		No discount rate	Deterministic	0.74
53	Tiwana	2012	US	Metabolic disorders	ICER	CEA	3d payer	Cohort-based study	Lifetime	All newborns	Current practice	Markov model	1 year		QALYs gained	3% (costs), 3% (health outcomes)	Deterministic	0.78
54	Hatam	2013	Iran	Glutaric aciduria Type 1	ICER	CEA	Societal	Cohort-based study	Not defined/Not clear	All newborns	No screening	Decision tree			QALYs gained	3% (costs), 3% (health outcomes)	Deterministic	0.71
55	Peterson	2013	US	(Critical) congenital heart disease	Not defined/Not clear	CEA	Healthcare	Cohort-based study	1 year	All newborns	No screening	Decision tree		Case detected		3% (health outcomes)	Deterministic, Probabilistic	0.75
56	Pfeil	2013	Germany	Glutaric aciduria type 1	ICER	CEA *	Healthcare	Cohort-based study	20/70 years	Not clear/Not described	No screening	Decision tree		Life-years gained	DALYs averted	No discount rate	Deterministic, Probabilistic	0.7

(*) These studies also included limited utility values.
